# Can Beneficial Microorganisms Reduce Phosphorus Application Rates in Common Bean (*Phaseolus vulgaris* L.) Cultivation in Sandy Soils?

**DOI:** 10.3390/plants15172555

**Published:** 2026-08-22

**Authors:** William Cesar Nishimoto Ito, Fernando Shintate Galindo, Guilherme Carlos Fernandes, Nelson Câmara de Souza Junior, Fernando de Souza Buzo, Karina da Silva Souza, Beatriz Silvério dos Santos, Mariana Cristina Barbosa, César Henrique Alves Seleguin, Thiago Assis Rodrigues Nogueira, Marcelo Carvalho Minhoto Teixeira Filho

**Affiliations:** 1 Department of Plant Health, Rural Engineering and Soil, Faculty of Engineering, Campus Ilha Solteira, São Paulo State University (UNESP), Ilha Solteira 15385-000, Brazil; william.nishimoto@unesp.br (W.C.N.I.); guilherme.carlos-fernandes@unesp.br (G.C.F.); souza.jr@unesp.br (N.C.d.S.J.); beatriz.silverio-santos@unesp.br (B.S.d.S.); mariana.c.barbosa@unesp.br (M.C.B.); cesar.seleguin@unesp.br (C.H.A.S.); mcm.teixeira-filho@unesp.br (M.C.M.T.F.); 2 Department of Plant Production (DPV), College of Agricultural and Technological Sciences, Campus Dracena, São Paulo State University (UNESP), Dracena 17900-000, Brazil; 3 Department of Agronomy, Campus Fernandópolis, Universidade Brasil, Fenandópolis 15613-899, Brazil; fsbuzo@gmail.com; 4 Department of Soil Science, School of Agricultural and Veterinary Sciences, Campus de Jaboticabal, São Paulo State University (UNESP), Jaboticabal 14884-900, Brazil; tar.nogueira@unesp.br

**Keywords:** *Phaseolus vulgaris*, *Trichoderma harzianum*, *Bacillus amyloliquefaciens*, P fertilization, grain yield, nutrient accumulation

## Abstract

Beneficial microorganisms enhance phosphorus (P) use efficiency in common bean, compensating for its shallow root system. Integrating biological approaches with reduced P rates optimizes plant growth, improves yield, lowers costs, and advances sustainable agriculture. This study aimed to assess the effects of seed inoculation with *Trichoderma harzianum* and/or *Bacillus amyloliquefaciens* associated with reduced P fertilization on common bean (*Phaseolus vulgaris* L.) agronomic and nutritional performance. The experiment was conducted under greenhouse conditions using a Quartzipsamment Entisol soil. Inoculation with *B. amyloliquefaciens* or *T. harzianum*, regardless of the P rate, as well as co-inoculation under the full phosphorus rate, increased grain yield by 64.4%, 31.9% and 49.4%, respectively, compared with the non-inoculated control under the full phosphorus rate. Regarding total phosphorus accumulation, inoculation with *T. harzianum* increased P accumulation by 90%, and co-inoculation increased it by 72%, compared with the non-inoculated control. The use of *B. amyloliquefaciens* and *T. harzianum* in common bean cultivation can enhance nutritional status (57% for total N accumulation, 40% for P, 93% for K, 33% for Ca, 31% for Mg, 50% for S) and grain productivity (44%), even with a 40% reduction in phosphorus fertilization in sandy soils with very low P availability, under greenhouse conditions.

## 1. Introduction

The common bean (*Phaseolus vulgaris* L.) belongs to the Fabaceae family and is among the most widely consumed foods worldwide. It plays a key role in food security due to its high contents of protein, carbohydrates, vitamins, dietary fiber, iron, and zinc [1]. In the 2023/2024 growing season, Brazilian common bean production reached 3.3 million tons, representing a 9.7% increase compared with the previous season, despite a reduced cultivated area of 2.4 million hectares [2]. Of this total, 2.85 million tons were destined for domestic consumption. Consequently, Brazil is recognized as one of the world’s largest producers and consumers of common bean [3].

In Brazil, *P. vulgaris* can be cultivated across three growing seasons, as the common bean is a short-cycle crop, which makes it a suitable option for crop rotation and contributes to its high profitability [4]. However, the species has a shallow and poorly developed root system, resulting in high sensitivity to soil conditions. Consequently, adequate physical, chemical, and biological soil properties are required, along with an efficient nutritional program and proper management of soil, pests, diseases, and weeds [5]. In this sense, according to data from MAPA, approximately 70% of the P fertilizers used in Brazil are imported, making the agricultural sector highly vulnerable to supply constraints and price fluctuations driven by global geopolitical and economic factors [6].

Phosphorus (P) is a macronutrient involved in nearly all biological processes in plants, acting either directly as a constituent of organic compounds or indirectly through ATP-mediated reactions [7,8,9]. Although required in smaller amounts than nitrogen, potassium, and calcium, phosphorus is often applied at higher rates because of its immobilization and low use efficiency in clay soils [10,11]. The limited availability of global P rock reserves highlights the need to improve phosphorus use efficiency (PUE) to ensure the sustainability of future agricultural production [12]. In this context, and in line with Sustainable Agriculture—one of the 17 Sustainable Development Goals [13]—the use of beneficial microorganisms represents a promising alternative for enhancing PUE, improving plant development, and increasing common bean productivity. These microorganisms, naturally associated with soils or plants, can be integrated into agricultural systems without disrupting ecological balance. Numerous studies have demonstrated their ability to influence plant growth, suppress pests and diseases, and increase crop yield [14,15,16].

Beneficial microorganisms, including fungi such as *Trichoderma harzianum* and bacteria such as *Rhizobium tropici*, *Bacillus* spp., and *Azospirillum brasilense*, can function as biostimulants by increasing the production of phytohormones, particularly auxins, which promote root system development and enhance nutrient uptake. They may also act as biofertilizers by solubilizing non-labile phosphorus via the release of organic acids, such as citric acid, thereby increasing its availability to plants, as well as by supplying other nutrients, including nitrogen, through biological fixation by bacteria [17]. In addition to improving fertilization efficiency, these microorganisms contribute to more sustainable agricultural systems.

*Bacillus amyloliquefaciens* is a beneficial bacterium that can function as both a biofertilizer and a biostimulant, with demonstrated potential to promote plant growth [18]. Studies involving common bean have shown that inoculation with this bacterium enhances plant growth and increases nutrient availability, particularly nitrogen and phosphorus, as well as pod formation and grain yield [19,20,21]. This happens because this bacterium is able to activate soil nutrients, altering their form and making them more available, as well as positively interfering with soil microbial communities, favoring absorption [19,20,21]. In research conducted with *Medicago sativa* L., *B. amyloliquefaciens* was effective in stimulating plant growth, leading to greater biomass accumulation, higher relative water content, increased chlorophyll levels, and improved gas exchange parameters under water stress conditions when compared with non-inoculated plants [22].

*Trichoderma harzianum* is a beneficial fungus and one of the most widely used agents in the biological control of plant diseases and nematodes [23]. In red kidney bean, this fungus improved the plant antioxidant enzyme activity and regulated the rhizosphere microbial community, resulting in increased plant growth and inhibition of root rot [24]. In addition, it has the ability to promote plant growth by acting as a biofertilizer, increasing nutrient availability, and by enhancing photosynthetic and respiratory efficiency in plants [25]. In maize, inoculation with *T. harzianum* has been shown to increase acid phosphatase activity, which is closely associated with phosphorus mobilization in tropical soils, as well as to raise phosphorus content in total plant dry matter [26]. In common bean cultivation, studies have demonstrated that inoculation with *T. harzianum* enhances root and shoot growth and improves nutrient uptake, resulting in higher productivity compared with non-inoculated plants [27,28].

Although many P-solubilizing strains exhibit broad-spectrum efficacy across diverse crops, the development of plant-specific, customized microbial coinoculation is poised to further optimize productivity by leveraging tailored rhizosphere interactions [29]. In this context, the study hypothesized that single inoculation and co-inoculation with *B. amyloliquefaciens* and *T. harzianum* exert a synergistic effect on the common bean rhizosphere by enhancing root architecture and P solubilization, thereby compensating for reduced P fertilization rates to maintain or enhance agronomic and nutritional performance. In addition, studies evaluating the effects of *T. harzianum* and *B. amyloliquefaciens* on common bean cultivated under reduced phosphorus rates remain limited. Therefore, this study aimed to assess the effects of two levels of phosphorus fertilization associated with inoculation with *B. amyloliquefaciens*, *T. harzianum*, their co-inoculation, and the control (no microorganisms) on the plant growth, yield potential, and nutritional status of common bean plants, under greenhouse conditions.

## 2. Results

### 2.1. Shoot Dry Mass, Root Dry Mass, and Grain Yield

In the evaluation of shoot dry mass (SDM) and root dry mass (RDM), no significant interaction was observed between inoculation and fertilization. However, significant main effects were detected: for SDM, differences occurred within both inoculation and fertilization factors, whereas for RDM, significant differences were observed only among inoculation treatments (Table 1 and Table S1).

For SDM, inoculation with beneficial microorganisms resulted in the highest values, with an average increase of approximately 56% compared with the control treatment. In addition, fertilization with 100% of the recommended P rate produced greater shoot dry mass per plant than the 60% application rate (Table 1).

Inoculation with *T. harzianum* alone and in combination with *B. amyloliquefaciens* resulted in the highest mean RDM values, followed by inoculation with *B. amyloliquefaciens*, whereas the non-inoculated treatment showed the lowest mean value. Treatments including *T. harzianum* exhibited an average increase of 119% in RDM compared with the control treatment (Table 1).

**Figure 1:**
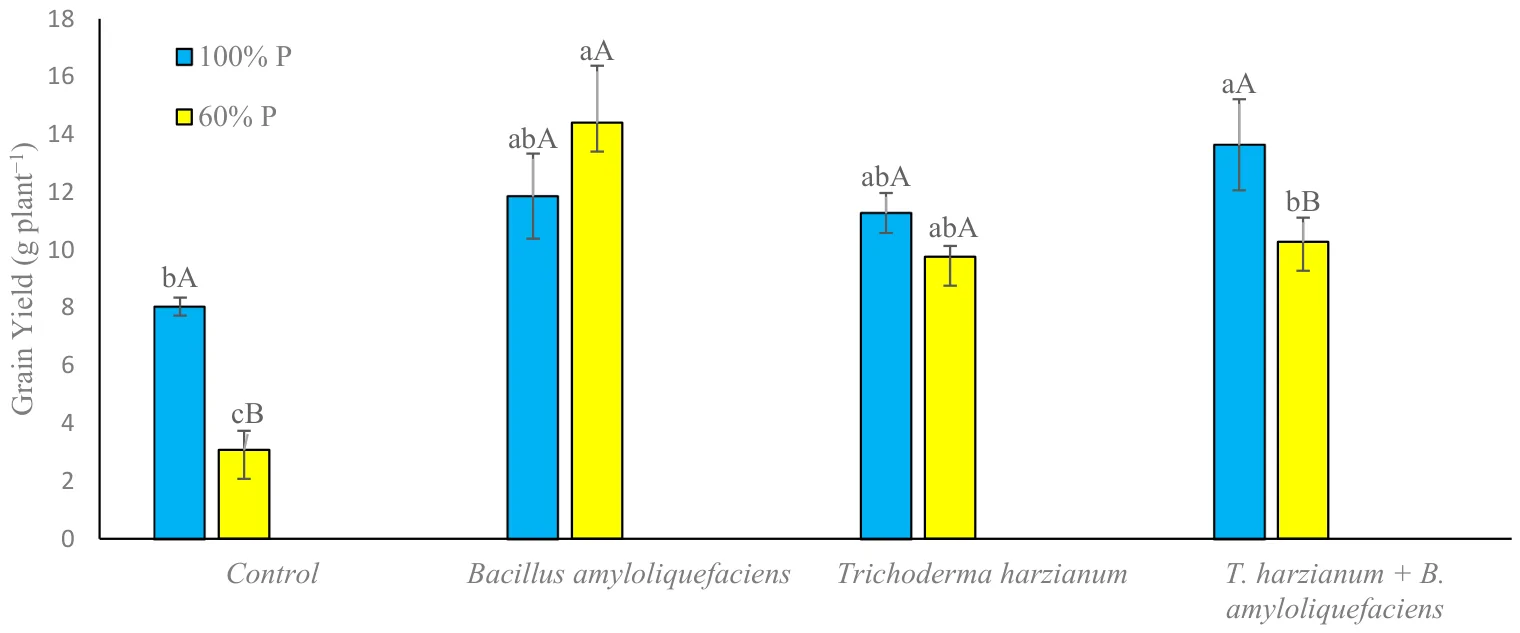
Grain yield of common bean (*Phaseolus vulgaris* L.) as affected by phosphorus application rates and inoculated seeds with beneficial microorganisms under greenhouse conditions. Different lowercase letters indicate significant differences among inoculation treatments within the same P rate, and different uppercase letters indicate significant differences among P rates within the same inoculation treatment, according to Tukey’s test (0.05 probability). Bars represent means ± Standard Error.

A significant interaction between inoculation and fertilization was observed for grain yield (GY) (Figure 1 and Table S1). Analysis of this interaction indicated that the non-inoculated treatment combined with the lowest P fertilization rate resulted in the lowest GY, with 3.06 g plant^−1^. For most inoculation treatments, regardless of the P rate applied (100% or 60%), the highest mean GY values were recorded, ranging from 10.2 to 14.4 g plant^−1^, except for the co-inoculation of *T. harzianum* + *B. amyloliquefaciens* at 60% of the recommended P rate. These values were substantially higher than those observed in the non-inoculated control at both phosphorus application rates.

### 2.2. Total Nutrient Accumulation

For total nutrient accumulation in common bean plants, no interaction between factors was observed for most nutrients; however, significant main effects were detected for individual accumulations, predominantly within the inoculation factor. Interaction effects between inoculation and fertilization were observed only for total calcium (Ca) accumulation (Table 2 and Table S2) and iron (Fe) accumulation (Table 3 and Table S2).

Significant differences among means were observed only for the inoculation factor with respect to total accumulation of nitrogen (N), phosphorus (P), potassium (K), sulfur (S), iron (Fe), manganese (Mn), copper (Cu), and zinc (Zn). For total magnesium (Mg) accumulation, statistically significant differences were detected independently for both inoculation and fertilization factors (Table 2 and Table 3).

The accumulation of N, K, Mg, and S (Table 2), as well as Mn, Cu, and Zn (Table 3), exhibited similar responses to inoculation. For all these nutrients, inoculated treatments showed higher mean values than the control, with average increases of 77% for N, 144% for K, 54% for Mg, 50% for S, 87% for Mn, 100% for Cu, and 60% for Zn. For phosphorus accumulation, inoculation with *T. harzianum* and co-inoculation with *T. harzianum* + *B. amyloliquefaciens* resulted in higher mean values compared with the control treatment (Table 2). P fertilization had an isolated effect only on total Mg accumulation, with the 60% recommended rate resulting in higher values than the 100% rate (Table 2).

**Figure 2:**
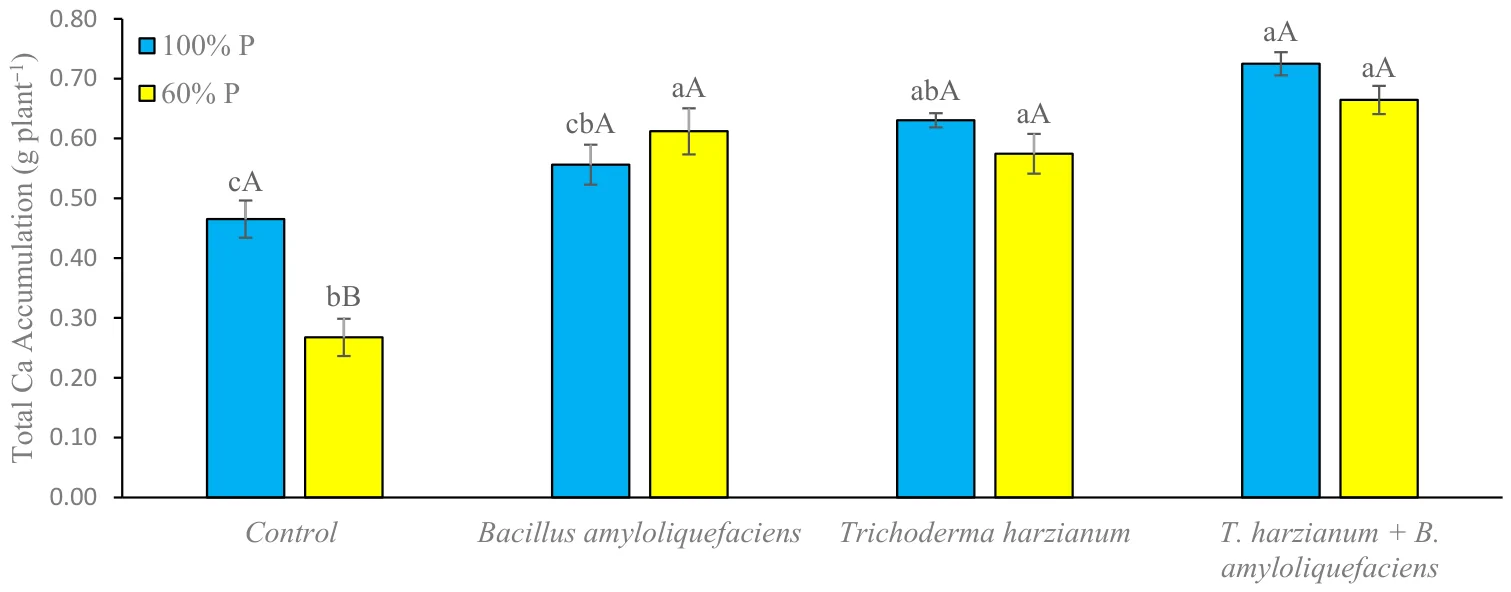
Total Ca accumulation in common bean (*Phaseolus vulgaris* L.) as affected by phosphorus application rates and inoculated seeds with beneficial microorganisms under greenhouse conditions. Different lowercase letters indicate significant differences among inoculation treatments within the same P rate, and different uppercase letters indicate significant differences among P rates within the same inoculation treatment, according to Tukey’s test (0.05 probability). Bars represent means ± Standard Error.

A significant interaction between inoculation and fertilization was observed for calcium (Ca) accumulation in common bean plants. The non-inoculated treatment combined with the lowest P_2_O_5_ rate resulted in the lowest mean values, whereas co-inoculation with *T. harzianum* + *B. amyloliquefaciens*, regardless of P fertilization level, and single inoculation with *T. harzianum* or *B. amyloliquefaciens* associated with the 60% rate produced the highest mean Ca accumulation values (Figure 2 and Supplementary Table S2).

**Figure 3:**
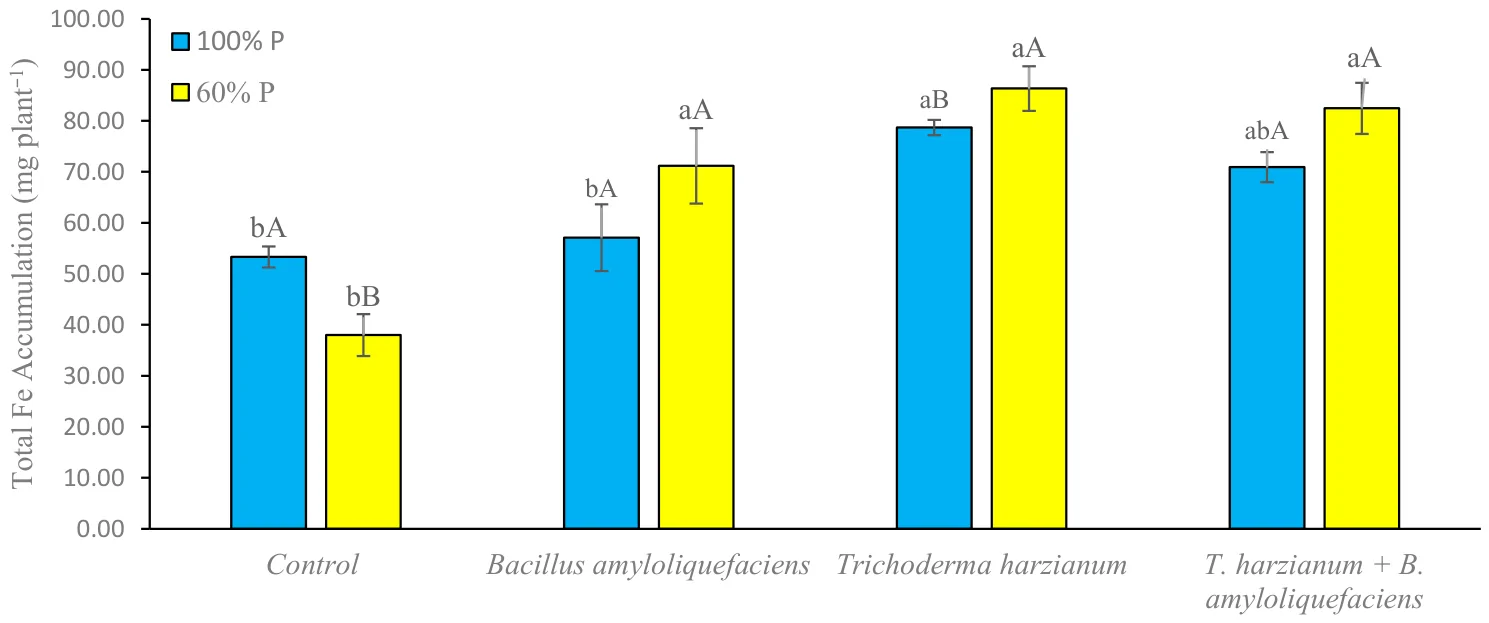
Total Fe accumulation in common bean (*Phaseolus vulgaris* L.) as affected by phosphorus application rates and inoculated seeds with beneficial microorganisms under greenhouse conditions. Different lowercase letters indicate significant differences among inoculation treatments within the same P rate, and different uppercase letters indicate significant differences among P rates within the same inoculation treatment, according to Tukey’s test (0.05 probability). Bars represent means ± Standard Error.

For the inoculation × fertilization (I × F) interaction affecting total iron (Fe) accumulation, the *T. harzianum* treatment combined with 60% of the recommended P rate showed the highest response and did not differ statistically from *B. amyloliquefaciens* at a 100% P rate or from the co-inoculation of *T. harzianum* + *B. amyloliquefaciens* at either 100% or 60% of the recommended P rate (Figure 3 and Supplementary Table S2).

## 3. Discussion

The use of microorganisms is an established practice in agriculture and can promote beneficial effects on plant growth and nutritional balance, thereby reducing the need for mineral fertilizers [30,31,32,33,34,35,36]. In the present study, the application of beneficial microorganisms clearly contributed to enhanced root and shoot growth and improved the nutritional status of common bean plants. Both the fungus *T. harzianum* and the bacterium *B. amyloliquefaciens*, applied individually or in co-inoculation, produced superior results compared with the control treatment, effects that may be associated with phytohormone production and phosphorus solubilization stimulated by these microorganisms [17,37,38,39].

Previous studies have demonstrated the ability of *T. harzianum* to produce the phytohormone auxin, which stimulates root system development by increasing root hair formation and, consequently, the root–soil contact area, thereby enhancing the efficiency of water and nutrient uptake [27,28,39], as also observed in the present experiment. With respect to *B. amyloliquefaciens*, the results of this study likewise indicate a biostimulatory effect on root system growth [18], as evidenced by the greater root dry mass compared with the control treatment.

These accumulation results can confirm the positive biostimulatory effects of microorganisms, as evidenced by the evaluations of RDM, SDM, and grain yield. The development of a larger root system, resulting from stimulated auxin production, increases the volume of soil explored by the roots, thereby enhancing water and nutrient uptake and leading to improved plant nutritional status and greater total nutrient accumulation.

Several studies have reported positive effects of beneficial microorganisms on the solubilization of phosphorus that is otherwise unavailable to plants [19,37,40,41]. In tropical agriculture, this solubilization of non-labile phosphorus is particularly important, as most soil P is rendered unavailable due to soil acidification and the presence of iron and aluminum oxides [40,41,42]. Nevertheless, proving a direct causal link between inoculation and P solubilization remains debatable. As Barrow and Lambers (2022) [43] noted, plant growth responses to “P-solubilizing” microorganisms may not stem from increased P supply, leaving it unclear whether benefits arise from soil P desorption or enhanced root uptake rates of the available pool.

The results of this study demonstrated that the use of microorganisms, applied individually or in combination, was able to maintain common bean productivity even at the lowest phosphorus application rate, while also ensuring adequate nutritional balance of nitrogen, phosphorus, potassium, and micronutrients. These findings confirm the efficiency of biological agents and highlight the need for further studies to elucidate their effects on plant metabolism, as well as for validation under field conditions.

The lower grain yield observed at the lowest phosphorus rate under co-inoculation of the two microorganisms may suggest reduced efficiency of this combination; however, repetition of the experiment would be necessary to confirm this response, as previous studies have reported positive effects of co-inoculation in different crops [36,44]. This result may also be related to the possible competition between microorganisms and plants under conditions of low P availability in the soil and low mineral P input, as microorganisms can be suppliers or competitors of P for plants, depending on the P status of the ecosystem [45,46].

P availability can affect microbial community structure, possibly exerting selective pressure on microbial communities and altering competitive interactions [47,48]. Therefore, when soil available P content is low, bacteria and fungi can compete for P, leading to the temporary immobilization of P within microbial biomass [49,50]. To overcome these limitations, future research must focus on enhancing the adaptability and persistence of functional strains in complex soil environments, alongside optimizing bioformulations and delivery systems [51].

These findings indicate that, in common bean, the use of the fungus alone has been reported to increase grain yield by approximately 10% to 25% [38], values lower than those obtained in the present study. Nonetheless, increases in grain yield ranging from 47% to 58% were observed with microorganism inoculation. These results demonstrate that farmers have access to effective tools for promoting more sustainable agricultural practices. Moreover, inoculation of common bean seeds with beneficial microorganisms, even under reduced P fertilization, can sustain plant growth and grain productivity comparable to or greater than those obtained with 100% P_2_O_5_ application in the absence of inoculation.

Overall, phosphorus application rates had limited influence on total nutrient accumulation, reinforcing the improved nutritional balance promoted by the use of microorganisms. Given the finite nature of P fertilizers, this approach represents an important component of more sustainable and regenerative agricultural systems, as it enhances the efficiency and utilization of non-labile phosphorus in soil dynamics, increases its availability to crops, and reduces reliance on mineral phosphorus inputs [19,37,38,52,53].

The data presented here and obtained in a greenhouse are promising, but further field research is needed to confirm their effectiveness under natural growing conditions. It is important to highlight that the two microorganisms with the best results in this study have a long history of research and positive outcomes. This is because these microorganisms help solubilize non-labile P in the soil, which is crucial for weathered tropical soils with high levels of Fe and Al oxides that exhibit a high P-fixation capacity. Furthermore, these microorganisms are responsible for greater mineralization of organic matter, making nutrients more available. All this reinforces the idea that both microorganisms can be important for the agricultural management of bean crops. The next steps should involve conducting this experiment in the field, preferably in different soil types, to verify the effectiveness of these two strains.

## 4. Materials and Methods

### 4.1. Study Area and Site Characterization

The study was conducted under greenhouse conditions in the municipality of Ilha Solteira, São Paulo State, Brazil, at approximately 51° 20′ 29″ W and 20° 25′ 06″ S. The soil used was classified as an Orthic Quartzarenic Neosol [54], corresponding to a Quartzipsamment Entisol according to Soil Taxonomy [55]. All pots were irrigated using a drip system. The controlled environmental conditions were as follows: (i) air temperature ranging from 25.2 ± 0.6 °C (minimum) to 35.8 ± 0.6 °C (maximum), with a mean of 30.5 ± 0.6 °C; (ii) relative air humidity between 60 and 65%.

### 4.2. Soil Sampling and Analysis

Prior to experiment establishment, disturbed soil (0–20 cm layer) was collected in the municipality of Selvíria, Mato Grosso do Sul, Brazil. After collection and transport, five disturbed individual soil samples were taken to form a composite sample and subsequently sent to the laboratory. Chemical characterization of the soil, performed according to the methodology described by Raij et al. (2001) [56], yielded the following results: resin-extractable P, 3 mg dm^−3^; S–SO_4_, 5 mg dm^−3^; organic matter, 15 g dm^−3^; pH (CaCl_2_), 5.4; K, Ca, Mg, and H + Al, 1.4, 10.0, 9.0, and 15.0 mmolc dm^−3^, respectively; Cu, Fe, Mn, and Zn (DTPA), 0.6, 17.0, 7.3, and 0.2 mg dm^−3^, respectively; B (hot water), 0.07 mg dm^−3^; cation exchange capacity (CEC), 35.4 mmolc dm^−3^; aluminum saturation, 0%; and base saturation, 58%.

### 4.3. Experimental Design and Treatments

The soil collected is characterized as “ravine soil”, which has been exposed to sunlight for a long period of time without any type of vegetation cover. This situation characterizes a soil with low microbial diversity and activity. Therefore, there was no need to carry out any disinfection process for this experiment.

The experiment was conducted using a randomized block design with five replications, arranged in a 4 × 2 factorial scheme, totaling 40 plots (plastic pots). The treatments consisted of four inoculation strategies: control without microorganism application, inoculation with *T. harzianum*, inoculation with *B. amyloliquefaciens*, and co-inoculation with *T. harzianum* + *B. amyloliquefaciens*, combined with two P application rates (26 and 44 kg ha^−1^) applied at sowing. The P application rates corresponded to 60% and 100% of the recommended dose for common bean cultivation, based on soil analysis and the fertilization guidelines proposed by Wutke et al. (2022) [57].

The selection of 60% of the recommended fertilization rate was strategically determined based on evidence that co-inoculation with beneficial microorganisms can offset mineral phosphorus requirements by up to 50% in soybean [58] and 50% in common bean [59] without compromising—and potentially enhancing—grain yields.

Microorganism inoculation was performed via seed treatment at sowing, using *T. harzianum* at a rate of 50 g ha^−1^ (isolate IBLF006, 1 × 10^10^ colony-forming units [CFU] g^−1^) and *B. amyloliquefaciens* at a rate of 200 mL ha^−1^ (strain CN-307, 1 × 10^8^ CFU mL^−1^). The application rates for both the fungus and the bacterium were calculated according to the manufacturer’s recommendations. The inoculants used were commercially available products registered with the Ministry of Agriculture, Livestock and Supply (MAPA) as *T. harzianum* isolate TRITTER^®^ and *B. amyloliquefaciens* strain N307^®^.

### 4.4. Experimental Setup and Management

On 15 September 2022, one week prior to sowing, each experimental unit—consisting of a 12 L plastic pot—was filled to capacity with soil. Based on soil analysis, liming was performed to raise base saturation to 70%, in accordance with Wutke et al. (2022) [57]. For liming, 3 g of dolomitic limestone (representing a rate of 500 kg ha^−1^; 86% RTNP) was used per pot, applying approximately 665 mg Calcium and 380 mg Magnesium per pot. Following liming, the pots were irrigated daily. Nitrogen, potassium, and the 100% phosphorus rate were established according to the expected yield of 3.0–4.0 t ha^−1^ and soil analysis, corresponding to 20 kg ha^−1^ of N, 83 kg ha^−1^ of K and 44 kg ha^−1^ of P [57].

At sowing, fertilization was applied directly in-furrow (via an X-shaped groove at a depth of 5 cm). Nitrogen and potassium were supplied using urea, 261 mg per pot (~120 mg N), and potassium chloride, 1000 mg per pot (~500 mg K). The 100% and 60% Phosphorus rates were supplied using triple superphosphate, 1333 mg per pot (~264 mg P) and 800 mg per pot (~158 mg P), respectively. In addition to NPK fertilization, sulfur, zinc, and boron were applied at rates of 3 kg ha^−1^ (zinc sulfate, supplying S and Zn, 18 mg per pot) and 1 kg ha^−1^ (boric acid, supplying B, 6 mg per pot).

The common bean cultivar used was IAC 1850, characterized by an indeterminate growth habit (type II) and a growth cycle of up to 90 days after sowing [60]. Four seeds were sown per pot, and all pots were irrigated to maintain soil moisture at 60% of water retention capacity, according to Galindo et al. (2025) [61], throughout the experimental period.

Twenty days after emergence (DAE), seedlings were thinned to maintain two plants per pot. At 30 DAE, nitrogen topdressing was applied in the form of urea, 1304 mg per pot (~587 mg N)—a rate equivalent to 100 kg N ha^−1^. Manual weed control was performed as required, and no chemical pesticides were applied for pest or disease management, as no infestations were observed during the experimental period. The experiment was harvested on 16 December 2022, completing a growth cycle of 85 days after sowing.

### 4.5. Measurements and Evaluations

Grain yield per plant: At physiological maturity (phenological stage R9), pods were harvested from each plant and manually threshed. Grain mass was determined and adjusted to 13% moisture content on a wet basis.

Shoot dry mass per plant: At harvest, shoots were cut and oven-dried at 65 °C for three days until constant weight was achieved, after which the samples were weighed.

Root dry mass per plant: Roots were separated from the shoots at the end of the growth cycle, washed, oven-dried at 65 °C for three days until constant weight, and then weighed.

Total nutrient accumulation: Dried grains, shoots, and root samples were ground using a Wiley-type knife mill for the determination of N, P, K, Ca, Mg, S, Fe, Mn, and Zn concentrations following the methodology described by Malavolta et al. (1997) [62]. Plant nutrient analysis involved sulfuric acid digestion for N determination—semi-micro Kjeldahl (brand: MARCONI, model MA036 plus, Piracicaba—Brasil) and nitric–perchloric digestion for the remaining elements. Phosphorus was measured via vanadate–molybdate spectrophotometry, S by turbidimetry in UV-VIS spectrophotometry (brand: FEMTO, model: 600 S, São Paulo—Brasil) and K, Ca, Mg, Fe, Mn, and Zn through atomic absorption spectrophotometry (brand: Agilent, model: 55B, Santa Clara, CA—USA). Total nutrient accumulation was calculated by multiplying the nutrient concentration in each plant component (grains, shoots, and roots) by the corresponding dry mass.

### 4.6. Statistical Analysis

Data analysis was conducted using R software (version 4.0.2) [63] with the ExpDes.pt experimental design package (version 1.2.2). The assumptions of normality and homoscedasticity were evaluated using the Shapiro–Wilk and Levene’s tests, respectively. Two-way analysis of variance (ANOVA) was subsequently performed, and the F-test was applied at the 1% and 5% probability levels to assess the effects of factors (Inoculations × P-rates) and their interactions. When significant differences were detected (*p* < 0.05), mean comparisons were conducted using Tukey’s test at the 5% probability level. The ANOVA was performed with 5 replications per treatment, and each replicate consisted of an average of 2 plants per pot (*n* = 5).

## 5. Conclusions

Our findings demonstrate that microbial inoculation and co-inoculation using *B. amyloliquefaciens* and *T. harzianum* significantly enhance nutrient accumulation, biomass allocation, and grain yield in *Phaseolus vulgaris*, under greenhouse conditions, cultivated in nutrient-poor sandy soils (Quartzipsamment Entisol). Crucially, this bio-based strategy mitigates the reliance on chemical inputs by enabling a 40% reduction in P fertilization, under controlled environments, without compromising crop productivity. These results are promising for future research and need to be validated under real field conditions.

## Figures and Tables

**Table 1:** Average values of grain yield (GY), shoot dry mass (SDM), and root dry mass (RDM) per common bean plant, as affected by phosphorus application rates and inoculated seeds with beneficial microorganisms under greenhouse conditions.

	GY	SDM	RDM
	g Plant^−1^		
**Inoculation**			
Control (without inoculation)	5.56 ± 0.90	19.4 ± 2.7 b	11.2 ± 0.8 c
*Trichoderma harzianum*	10.52 ± 0.45	29.0 ± 2.1 a	25.1 ± 1.5 a
*Bacillus amyloliquefaciens*	13.13 ± 1.24	29.0 ± 1.0 a	19.2 ± 1.2 b
*T. harzianum + B. amyloliquefaciens*	11.96 ± 1.01	32.8 ± 2.3 a	23.9 ± 0.8 a
**P-fertilization**			
100% recommended dose	11.21 ± 0.70	30.7 ± 1.5 a	19.2 ± 1.2
60% recommended dose	9.38 ± 1.07	24.3 ± 1.8 b	20.4 ± 1.7
**F-test**	*p*-value		
Inoculation (I)	0.0000 **	0.0001 **	0.0000 **
Fertilization (F)	0.0192 *	0.0013 **	0.6987 ^ns^
I × F	0.0077 **	0.0605 ^ns^	0.2318 ^ns^
**Overall Average**	10.29	27.55	19.82
**CV (%)**	24.88	20.59	18.53

**Note:** GY: grain yield; SDM: shoot dry matter; RDM: root dry matter; CV (%): coefficient of variation. Means followed by different letters in the column for different treatments differ according to Tukey’s test at 5% probability. **: significant at 1% probability; *: significant at 5% probability; ^ns^: not significant.

**Table 2:** Average values of total macronutrient accumulation per common bean plant, as affected by phosphorus application rates and inoculated seeds with beneficial microorganisms under greenhouse conditions.

	N	P	K	Ca	Mg	S
	g Plant^−1^					
**Inoculation**						
Control	0.56 ± 0.08 b	0.11 ± 0.02 b	0.31 ± 0.05 b	0.37 ± 0.04	0.26 ± 0.02 b	0.10 ± 0.01 b
*T. harzianum*	1.02 ± 0.07 a	0.21 ± 0.02 a	0.66 ± 0.06 a	0.60 ± 0.02	0.38 ± 0.03 a	0.16 ± 0.01 a
*B. amyloliquefaciens*	1.04 ± 0.07 a	0.17 ± 0.02 ab	0.66 ± 0.07 a	0.58 ± 0.03	0.37 ± 0.02 a	0.13 ± 0.01 a
*T. harzianum* + *B. amyloliquefaciens*	1.17 ± 0.05 a	0.19 ± 0.02 a	0.76 ± 0.07 a	0.69 ± 0.02	0.45 ± 0.02 a	0.16 ± 0.01 a
**P-dose fertilization**						
100% recommended	0.97 ± 0.06	0.18 ± 0.01	0.60 ± 0.05	0.59 ± 0.03	0.31± 0.02 b	0.14 ± 0.01
60% recommended	0.93 ± 0.09	0.17 ± 0.02	0.60 ± 0.07	0.53 ± 0.04	0.42± 0.03 a	0.14 ± 0.01
**F-Test**	*p*-value					
Inoculation (I)	0.0000 **	0.0049 **	0.0000 **	0.0000 **	0.003 **	0.000 **
Fertilization (F)	0.6062 ^ns^	0.6853 ^ns^	0.9975 ^ns^	0.0045 **	0.0016 **	0.8276 ^ns^
I × F	0.2854 ^ns^	0.6237 ^ns^	0.1570 ^ns^	0.0027 **	0.3205 ^ns^	0.3708 ^ns^
**Overall Average**	0.95	0.17	0.61	0.56	0.36	0.14
**CV (%)**	21.36	29.56	22.56	10.24	19.62	17.45

**Note:** CV (%): coefficient of variation. Means followed by different letters in the column for different treatments differ according to Tukey’s test at 5% probability. **: significant at 1% probability; ^ns^: not significant.

**Table 3:** Average values of total micronutrient accumulation in common bean per common bean plant, as affected by phosphorus application rates and inoculated seeds with beneficial microorganisms under greenhouse conditions.

	Fe	Mn	Cu	Zn
	mg Plant^−1^			
**Inoculation**				
Control (no inoculation)	45.6 ± 3.5	1.92 ± 0.26 b	0.84 ± 0.11 b	1.52 ± 0.17 b
*Trichoderma harzianum*	82.5 ± 2.4	3.82 ± 0.20 a	1.36 ± 0.11 a	2.36 ± 0.15 a
*Bacillus amyloliquefaciens*	64.1 ± 5.5	3.02 ± 0.26 a	1.68 ± 0.20 a	2.25 ± 0.14 a
*T. harzianum + B. amyloliquefaciens*	76.7 ± 3.4	3.89 ± 0.27 a	1.66 ± 0.07 a	2.31 ± 0.10 a
**P-fertilizantion**				
100% recommended dose	65.0 ± 3.0	3.08 ± 0.25	1.33 ± 0.14	2.20 ± 0.14
60% recommended dose	69.5 ± 5.4	3.24 ± 0.28	1.44 ± 0.10	2.01 ± 0.12
**F-Test**	*p*-value			
Inoculation (I)	0.0000 **	0.0000 **	0.0002 **	0.0008 **
Fertilization (F)	0.2151 ^ns^	0.5275 ^ns^	0.4008 ^ns^	0.1814 ^ns^
I × F	0.0284 *	0.6017 ^ns^	0.1338 ^ns^	0.9685 ^ns^
**Overall Average**	67.25	3.16	1.39	2.11
**CV (%)**	14.78	21.99	24.60	18.33

**Note:** CV (%): coefficient of variation. Means followed by different letters in the column for different treatments differ according to Tukey’s test at 5% probability. **: significant at 1% probability; *: significant at 5% probability; ^ns^: not significant.

## Data Availability

The original contributions presented in this study are included in the article. Further inquiries can be directed to the corresponding author.

## Supplementary Materials

The following supporting information can be downloaded at: https://www.mdpi.com/article/10.3390/plants15172555/s1, Supplementary Table S1. Average values of grain yield (GY), shoot dry mass (SDM), and root dry mass (RDM) per common bean plant, as affected by the interaction of phosphorus application rates and inoculated seeds with beneficial microorganisms under greenhouse conditions. Supplementary Table S2. Average values of total nutrient accumulation per common bean plant, as affected by the interaction of phosphorus application rates and inoculated seeds with beneficial microorganisms under greenhouse conditions.

## Abbreviations

The following abbreviations are used in this manuscript:

GY	Grain Yield
RDM	Root Dry Mass
RTNP	Relative Total Neutralizing Power
SDM	Shoot Dry Mass

## References

[1] Domene S.M.Á., Ghedini N.S.R.V., Steluti J. (2021). Importância Nutricional Do Arroz e Do Feijão. Arroz e Feijão: Tradição e Segurança Alimentar.

[2] Companhia Nacional de Abastecimento-Conab (2023). Acompanhamento Da Safra Brasileira de Grãos Safra 2022/23.

[3] Coêlho J.D., Ximenes L.F. (2020). Feijão: Produção e Mercado. Cad. Setorial Etene.

[4] da SIlva M.G. (2015). Implantação Da Cultura. Aspectos Gerais da Cultura do Feijão Phaseolus vulgaris L.

[5] Carvalho M.C.S., Silveira P.M. (2021). Adubação. In: Cultivo de Feijão. Publicações EMBRAPA: Embrapa Arroz e Feijão. https://www.embrapa.br/agencia-deinformacao-tecnologica/cultivos/feijao/producao/adubacao.

[6] Polidoro J.C. (2025). Cenário da produção nacional de fertilizantes no Brasil 2025–2030: Ações estratégicas para o aumento da produção nacional. Brasília: Ministério da Agricultura e Pecuária/Embrapa. https://www.gov.br/agricultura/pt-br/assuntos/insumos-agropecuarios/insumos-agricolas/fertilizantes/plano-nacional-de-fertilizantes.

[7] Prado R.D.M. (2008). Nutrição De Plantas.

[8] Taiz L., Zeiger E. (2017). Fisiologia Vegetal.

[9] Nesme T., Metson G.S., Bennett E.M. (2018). Global Phosphorus Flows through Agricultural Trade. Glob. Environ. Change.

[10] Silva L.I.D., Pereira M.C., Carvalho A.M.X.D., Buttrós V.H., Pasqual M., Dória J. (2023). Phosphorus-Solubilizing Microorganisms: A Key to Sustainable Agriculture. Agriculture.

[11] Costa K.S.D.Q., Oliveira C.F.D., Melo M.P., Vaz C.F., Melo N.C., Moraes F.K.C. (2024). Fósforo No Sistema Solo-Planta: Uma Revisão. Obs. Econ. Latinoam..

[12] Yu X., Keitel C., Dijkstra F.A. (2021). Global Analysis of Phosphorus Fertilizer Use Efficiency in Cereal Crops. Glob. Food Secur..

[13] Organização das Nações Unidas (ONU) (2015). Transformando Nosso Mundo: A Agenda 2030 para o Desenvolvimento Sustentável. Nova York: ONU. https://brasil.un.org/sites/default/files/2020-09/agenda2030-pt-br.pdf.

[14] Gomes E.A., Silva U.D.C., Paiva C.A.D.O., Lana U.G.D.P., Marriel I.E., Santos V.L. (2016). Microrganismos Promotores Do Crescimento de Plantas.

[15] Ganugi P., Martinelli E., Lucini L. (2021). Microbial Biostimulants as a Sustainable Approach to Improve the Functional Quality in Plant-Based Foods: A Review. Curr. Opin. Food Sci..

[16] Elnahal A.S.M., El-Saadony M.T., Saad A.M., Desoky E.-S.M., El-Tahan A.M., Rady M.M., AbuQamar S.F., El-Tarabily K.A. (2022). The Use of Microbial Inoculants for Biological Control, Plant Growth Promotion, and Sustainable Agriculture: A Review. Eur. J. Plant Pathol..

[17] Lopes M.J.D.S., Santiago B.S., Silva I.N.B.D., Gurgel E.S.C. (2021). Biotecnologia Microbiana: Inoculação, Mecanismos de Ação e Benefícios Às Plantas. Res. Soc. Dev..

[18] Luo L., Zhao C., Wang E., Raza A., Yin C. (2022). Bacillus Amyloliquefaciens as an Excellent Agent for Biofertilizer and Biocontrol in Agriculture: An Overview for Its Mechanisms. Microbiol. Res..

[19] Sabaté D.C., Brandán C.P. (2022). Bacillus Amyloliquefaciens Strain Enhances Rhizospheric Microbial Growth and Reduces Root and Stem Rot in a Degraded Agricultural System. Rhizosphere.

[20] Anzuma A., Hossain M.M., Mohi-Ud-Din M., Nazran A., Khan H.I., Islam S.M.N., Ghosh T.K. (2024). Enhancing Drought Tolerance in Common Bean by Plant Growth Promoting Rhizobacterium *Bacillus amyloliquefaciens*. Acta Agric. Slov..

[21] Yang P., Yuan P., Liu W., Zhao Z., Bernier M.C., Zhang C., Adhikari A., Opiyo S.O., Zhao L., Banks F. (2024). Plant Growth Promotion and Plant Disease Suppression Induced by *Bacillus amyloliquefaciens* Strain GD4a. Plants.

[22] Han L., Zhang M., Du L., Zhang L., Li B. (2022). Effects of Bacillus Amyloliquefaciens QST713 on Photosynthesis and Antioxidant Characteristics of Alfalfa (*Medicago Sativa* L.) under Drought Stress. Agronomy.

[23] Meyer M.C., Mazaro S.M., da Silva J.C. (2019). Trichoderma: Uso na agricultura.

[24] Guo Z., Zhang J., Liu Z., Li Y., Li M., Meng Q., Yang Z., Luo Y., Zhang Q., Yan M. (2024). Trichoderma Harzianum Prevents Red Kidney Bean Root Rot by Increasing Plant Antioxidant Enzyme Activity and Regulating the Rhizosphere Microbial Community. Front. Microbiol..

[25] Pani S., Kumar A., Sharma A. (2021). Trichoderma Harzianum: An Overview. Bull. Environ. Pharmacol. Life Sci..

[26] De Oliveira H.P., De Melo R.O., Cavalcante V.S., Monteiro T.S.A., De Freitas L.G., Lambers H., Valadares S.V. (2025). Phosphate Fertilizers Coated with Phosphate-Solubilising Trichoderma Harzianum Increase Phosphorus Uptake and Growth of Zea Mays. Plant Soil.

[27] Mahmoodian S., Kowsari* M., Motallebi M., Zamani M., Jahromi Z.M. (2022). Effect of Improved Trichoderma Harzianum on Growth and Resistance Promotion in Bean Plant. Braz. Arch. Biol. Technol..

[28] Battaglia M.E., Martinez S.I., Covacevich F., Consolo V.F. (2024). *Trichoderma harzianum* Enhances Root Biomass Production and Promotes Lateral Root Growth of Soybean and Common Bean under Drought Stress. Ann. Appl. Biol..

[29] Timofeeva A., Galyamova M., Sedykh S. (2022). Prospects for Using Phosphate-Solubilizing Microorganisms as Natural Fertilizers in Agriculture. Plants.

[30] Kour D., Rana K.L., Yadav A.N., Yadav N., Kumar M., Kumar V., Vyas P., Dhaliwal H.S., Saxena A.K. (2020). Microbial Biofertilizers: Bioresources and Eco-Friendly Technologies for Agricultural and Environmental Sustainability. Biocatal. Agric. Biotechnol..

[31] Nosheen S., Ajmal I., Song Y. (2021). Microbes as Biofertilizers, a Potential Approach for Sustainable Crop Production. Sustainability.

[32] Galindo F.S., Pagliari P.H., Fernandes G.C., Rodrigues W.L., Boleta E.H.M., Jalal A., Céu E.G.O., Lima B.H.D., Lavres J., Teixeira Filho M.C.M. (2022). Improving Sustainable Field-Grown Wheat Production With Azospirillum Brasilense Under Tropical Conditions: A Potential Tool for Improving Nitrogen Management. Front. Environ. Sci..

[33] Gaspareto R.N., Jalal A., Ito W.C.N., Oliveira C.E.D.S., Garcia C.M.D.P., Boleta E.H.M., Rosa P.A.L., Galindo F.S., Buzetti S., Ghaley B.B. (2023). Inoculation with Plant Growth-Promoting Bacteria and Nitrogen Doses Improves Wheat Productivity and Nitrogen Use Efficiency. Microorganisms.

[34] Erdin F., Kulaz H. (2025). The Effect of Inorganic and Microbial Fertilizer Applications on the Grain Yield and Some Agronomic Characters of the Bean (*Phaseolus vulgaris* L.)^##^. LEGUME Res.-Int. J..

[35] Fortu S.T., Tening A.S., Ndzeshala S.T., Ngosong C. (2025). Exploring the Potential of Microbial Inoculant to Enhance Common Bean (*Phaseolus vulgaris* L:) Yield via Increased Root Nodulation and Soil Macro-Nutrients. PLoS ONE.

[36] Oliveira T.M., Cardozo Gonçalves Cantão V., Matias De Oliveira A., Oliveira Santos G., Francischini R., Freire E.S., Moraes Tavares R.L., Morais L.K.D.O., Duarte W.T.F., Santos E.Z.D. (2025). *Bacillus amyloliquefaciens* and *Trichoderma asperellum* in Association with Humic Substances to Promote the Growth of Soybean Plants. J. Plant Nutr..

[37] Sheteiwy M.S., Abd Elgawad H., Xiong Y., Macovei A., Brestic M., Skalicky M., Shaghaleh H., Alhaj Hamoud Y., El-Sawah A.M. (2021). Inoculation with *Bacillus amyloliquefaciens* and Mycorrhiza Confers Tolerance to Drought Stress and Improve Seed Yield and Quality of Soybean Plant. Physiol. Plant..

[38] De Souza L.F.R., De Souza Júnior N.C., Fernandes G.C., Ito W.C.N., Barbosa M.C., Freitas L.B., Da Silva Souza K., Alexandre L.D.S., Silva M.B., Da Silva E.C. (2024). Response of *Phaseolus vulgaris* to the Use of Growth-Promoting Microorganisms Associated with the Reduction of NPK Fertilization in Tropical Soils: Clayey Oxisol and Sandy Ultisol. Agriculture.

[39] El-Sharkawy H.H.A., Rashad Y.M., El-Blasy S.S.A., Amin B.H., Youssef M.A.A., Hafez M., Abd-ElGawad A., Bourouah M., Elsherbiny E.A., Yousef S.A. (2025). Mycorrhizal Symbiosis and Application of Vitamin B3-Treated Trichoderma Harzianum HE24 Additively Trigger Immunity Responses in Faba Bean Plants against Rhizoctonia Root Rot and Promote the Plant Growth and Yield. BMC Plant Biol..

[40] Rawat P., Shankhdhar D., Shankhdhar S.C. (2022). Synergistic Impact of Phosphate Solubilizing Bacteria and Phosphorus Rates on Growth, Antioxidative Defense System, and Yield Characteristics of Upland Rice (*Oryza Sativa* L.). J. Plant Growth Regul..

[41] Boitt G., Schmitt D.E., Gatiboni L.C., Wakelin S.A., Black A., Sacomori W., Cassol P.C., Condron L.M. (2018). Fate of Phosphorus Applied to Soil in Pig Slurry under Cropping in Southern Brazil. Geoderma.

[42] Barbosa M.F., Sales R.M.M., Galarza F.A.D., Kruger C.Q., Fávaro L.C.D.L., Quirino B.F. (2025). Biological Resources Driving Productivity: Bioinputs for Sustainable Plant Agriculture in Brazil. Sustain. Microbiol..

[43] Barrow N.J., Lambers H. (2022). Phosphate-Solubilising Microorganisms Mainly Increase Plant Phosphate Uptake by Effects of pH on Root Physiology. Plant Soil.

[44] Karan A., Mishra U., Kumari S., Khushi, Khatri S., Singh A. (2025). Biostimulatory Role of Bacillus Amyloliquefaciens AUPPB02 in Barley: From In-Vitro Characterization to Pot Trials. Plant Arch..

[45] Turner B.L., Lambers H., Condron L.M., Cramer M.D., Leake J.R., Richardson A.E., Smith S.E. (2013). Soil Microbial Biomass and the Fate of Phosphorus during Long-Term Ecosystem Development. Plant Soil.

[46] Richardson A.E., Simpson R.J. (2011). Soil Microorganisms Mediating Phosphorus Availability Update on Microbial Phosphorus. Plant Physiol..

[47] Liu H., Li S., Qiang R., Lu E., Li C., Zhang J., Gao Q. (2022). Response of Soil Microbial Community Structure to Phosphate Fertilizer Reduction and Combinations of Microbial Fertilizer. Front. Environ. Sci..

[48] Benmrid B., Ghoulam C., Zeroual Y., Kouisni L., Bargaz A. (2023). Bioinoculants as a Means of Increasing Crop Tolerance to Drought and Phosphorus Deficiency in Legume-Cereal Intercropping Systems. Commun. Biol..

[49] Luo Y., Ma L., Feng Q., Luo H., Chen C., Wang S., Yuan Y., Liu C., Cao X., Li N. (2024). Influence and Role of Fungi, Bacteria, and Mixed Microbial Populations on Phosphorus Acquisition in Plants. Agriculture.

[50] Peñuelas J., Zheng B., Tariq A., Sardans J. (2026). Microbial Phosphorus Cycling in Terrestrial Ecosystems. Nat. Rev. Microbiol..

[51] Zhu Y., Pan F., Ji C., Zhou B., Li M., Shi W., Gao Z. (2026). Critical Role of Microorganisms in Phosphorus Cycling of Agroecosystems within the Plant-Soil Interface. Plant Soil.

[52] Hegyi A., Nguyen T.B.K., Posta K. (2021). Metagenomic Analysis of Bacterial Communities in Agricultural Soils from Vietnam with Special Attention to Phosphate Solubilizing Bacteria. Microorganisms.

[53] Alves Ribeiro N.A., Silva Matos A.M., Modesto V.C., De Souza Júnior N.C., Moreira Girardi V.A., Mendes I.D.C., Andreotti M. (2025). Soil Bioindicators and Crop Productivity Affected by Legacy Phosphate Fertilization and *Azospirillum Brasilense* Inoculation in No-Till Systems. Appl. Sci..

[54] Santos H.G., Jacomina P.K.T., Dos Anjos L.H.C., De Oliveira V.A., Lumbreras J.F., Coelho M.R., De Almeida J.A., De Araujo Filho J.C., De Oliveira J.B., Cunha T.J.F. (2018). Brazilian Soil Classification System.

[55] Soil Survey Staff (2014). Keys to Soil Taxonomy.

[56] van Raij B., Andrade J.C., Cantarella H., Quaggio J.A. (2001). Chemical Analysis for Fertility Evaluation of Tropical Soils.

[57] Wutke E.B., Chiorato A.F., Esteves J.A.d.F., Carbonell S.A.M., Ambrosano E.J., Lemos L.B., Soratto R.P., Arf O., Cantarella H. (2022). FEIJÃO (*Phaseolus vulgaris*). BOLETIM 100: RECOMIENDAÇÕES DE ADUBAÇÃO EE CALAGEM PARA O ESTADO DE SÃO PAULO.

[58] Oliveira Paiva C.A., Alves V.M.C., Gomes E.A., Tinoco S.M.D.S., Lana U.G.D.P., Marrie I.E. (2022). Microrganismos Solubilizadores de Fósforo e Potássio Na Cultura Da Soja. Bioinsumos na cultura da soja.

[59] Bittencourt C.D., Messias M., Wendland A., De Brito Ferreira E.P. (2024). Phosphate-Solubilizing Inoculant Improves Agronomic Performance of Common Bean with Reduced Phosphate Fertilizer Dose. J. Soil Sci. Plant Nutr..

[60] Carbonell S.A.M., Chiorato A.F., Bezerra L.M.C., Gonçalves J.G.R., Silva D.A.D., Esteves J.A.D.F., Benchimol-Reis L.L., Carvalho C.R.L., Barros V.L.N.P.D., Freitas R.S.D. (2019). IAC 1850: High Yielding Carioca Common Bean Cultivar. Crop Breed. Appl. Biotechnol..

[61] Galindo F.S., Thiengo C.C., Pagliari P.H., Bernardes J.V.S., Dos Santos G.D., Longato P.A.F., De Sousa Vilela L., Teixeira Filho M.C.M., Azevedo R.A., Gaziola S.A. (2025). Interactive Effects of Bacterial Consortia and Basal Nitrogen Fertilization on Initial Maize Growth: An Investigation Based on Physiological Parameters and 15N Isotopic Analysis. J. Plant Growth Regul..

[62] Malavolta E., Vitti G.C., Oliveira S.O. (1997). Evaluation of the Nutritional Status of Plants: Principles and Applications.

[63] R Studio Team (2019). RStudio: Integrated Development for R 2019.

