# Supplementary material for: Can Beneficial Microorganisms Reduce Phosphorus Application Rates in Common Bean (Phaseolus vulgaris L.) Cultivation in Sandy Soils?
Source: Plants (Basel). 2026 Aug 22;15(17):2555. doi: 10.3390/plants15172555 (PMC13567017; doi:10.3390/plants15172555)
Supplement: Supplementary file 1 [file plants-15-02555-s001.zip › plants-4442437-supplementary.pdf]

Supplementary Table S1. Average values of grain yield (GY), shoot dry mass (SDM), and root dry mass (RDM) per common bean plant, as affected by the interaction of phosphorus application rates and inoculated seeds with beneficial microorganisms under greenhouse conditions.

Grain Yield (g plant^{-1})		
	P – fertilization (F)	
**Inoculation (I)**	100%	60%
Control (no inoculation)	8.0 ± 0.31 bA	3.1 ± 0.66 cB
Trichoderma harzianum	11.3 ± 0.69 abA	9.8 ± 0.38 bA
Bacillus amyloliquefaciens	11.9 ± 1.47 abA	14.4 ± 1.97 aA
T. harzianum + B. amyloliquefaciens	13.6 ± 1.57 aA	10.3 ± 0.83 bB
**F-value (IxF)**	4.842	
**P-value (IxF)**	0.0077 **	

Shoot Dry Mass (g plant^{-1})		
	P – fertilization (F)	
**Inoculation (I)**	100%	60%
Control (no inoculation)	25.8 ± 3.53 bA	13.4 ± 0.60 bB
Trichoderma harzianum	31.4 ± 2.23 abA	26.6 ± 3.52 aA
Bacillus amyloliquefaciens	28.4 ± 1.50 abA	29.6 ± 1.43 aA
T. harzianum + B. amyloliquefaciens	37.4 ± 2.66 aA	27.8 ± 2.37 aB
**F-value (IxF)**	2,765	
**P-value (IxF)**	0,0605 ns	

Root Dry Mass (g plant^{-1})		
	P – fertilization (F)	
**Inoculation (I)**	100%	60%
Control (no inoculation)	13.0 ± 0.89 bA	9.4 ± 0.87 bA
Trichoderma harzianum	24.6 ± 1.47 aA	25.6 ± 2.79 aA
Bacillus amyloliquefaciens	18.4 ± 1.69 abA	19.8 ± 1.91 aA
T. harzianum + B. amyloliquefaciens	22.4 ± 1.21 aA	25.4 ± 0.81 aA
**F-value (IxF)**	1.517	
**P-value (IxF)**	0.2318 ns	

Note:. *: $p < 0.05$, **: $p < 0.01$, ns: not significant. Different lowercase letters indicate significant differences among inoculation treatments within the same P rate, and different uppercase letters indicate significant differences among P rates within the same inoculation treatment, according to Tukey's test (0.05 probability).

Supplementary Table S2. Average values of total nutrient accumulation per common bean plant, as affected by the interaction of phosphorus application rates and inoculated seeds with beneficial microorganisms under greenhouse conditions.

**Total N Accumulation (g plant^{-1})**		
	**P – fertilization (F)**	
**Inoculation (I)**	100%	60%
Control (no inoculation)	0.7 ± 0.11 bA	0.42 ± 0.06bA
Trichoderma harzianum	1.05 ± 0.13 abA	1.02 ± 0.08 aA
Bacillus amyloliquefaciens	0.97 ± 0.08 abA	1.07 ± 0.12 aA
T. harzianum + B. amyloliquefaciens	1.15 ± 0.09 aA	1.20 ± 0.07 aA
**F-value (IxF)**	1.349	
**P-value (IxF)**	0.2854 ^{ns}	

**Total P Accumulation (g plant^{-1})**		
	**P – fertilization (F)**	
**Inoculation (I)**	100%	60%
Control (no inoculation)	0.14 ± 0.03 aA	0.09 ± 0.03 bA
Trichoderma harzianum	0.21 ± 0.03 aA	0.22 ± 0.02 aA
Bacillus amyloliquefaciens	0.16 ± 0.01 aA	0.18 ± 0.04 abA
T. harzianum + B. amyloliquefaciens	0.20 ± 0.03 aA	0.19 ± 0.01 aA
**F-value (IxF)**	0.597	
**P-value (IxF)**	0.6237 ^{ns}	

**Total K Accumulation (g plant^{-1})**		
	**P – fertilization (F)**	
**Inoculation (I)**	100%	60%
Control (no inoculation)	0.37 ± 0.06 bA	0.25 ± 0.06 bA
Trichoderma harzianum	0.70 ± 0.07 aA	0.62 ± 0.10 aA
Bacillus amyloliquefaciens	0.57 ± 0.07 abA	0.75 ± 0.10 aA
T. harzianum + B. amyloliquefaciens	0.75 ± 0.09aA	0.77 ± 0.12 aA
**F-value (IxF)**	1.922	
**P-value (IxF)**	0.1570 ^{ns}	

**Total Ca Accumulation (g plant^{-1})**		
	**P – fertilization (F)**	
**Inoculation (I)**	100%	60%
Control (no inoculation)	0.46 ± 0.03 cA	0.27 ± 0.03 bB
Trichoderma harzianum	0.63 ± 0.01 abA	0.57 ± 0.03 aA
Bacillus amyloliquefaciens	0.56 ± 0.03 cbA	0.61 ± 0.04 aA
T. harzianum + B. amyloliquefaciens	0.72 ± 0.02 aA	0.66 ± 0.02 aA
**F-value (IxF)**	6.514	
**P-value (IxF)**	0.0027 **	

**Total Mg Accumulation (g plant^{-1})**		
	**P – fertilization (F)**	
**Inoculation (I)**	100%	60%
Control (no inoculation)	0.23 ± 0.03 aA	0.28 ± 0.04 bA
Trichoderma harzianum	0.35 ± 0.05 aA	0.41 ± 0.02 abA

Bacillus amyloliquefaciens	0.34 ± 0.02 aA	0.40 ± 0.04 abA
T. harzianum + B. amyloliquefaciens	0.36 ± 0.05 aB	0.53 ± 0.02 aA
**F-value (IxF)**	1.239	
**P-value (IxF)**	0.3205 ns	

**Total S Accumulation (g plant^{-1})**		
	**P – fertilization (F)**	
**Inoculation (I)**	100%	60%
Control (no inoculation)	0.10 ± 0.02 bA	0.09 ± 0.01 bA
Trichoderma harzianum	0.17 ± 0.01 aA	0.15 ± 0.01 aA
Bacillus amyloliquefaciens	0.12 ± 0.01 abA	0.14 ± 0.01 aA
T. harzianum + B. amyloliquefaciens	0.16 ± 0.01 aA	0.16 ± 0.01 aA
**F-value (IxF)**	1.101	
**P-value (IxF)**	0.3708 ns	

**Total Fe Accumulation (mg plant^{-1})**		
	**P – fertilization (F)**	
**Inoculation (I)**	100%	60%
Control (no inoculation)	53.3 ± 2.1 bA	38.0 ± 4.1 bB
Trichoderma harzianum	78.7 ± 1.5 aA	86.3 ± 4.4 aA
Bacillus amyloliquefaciens	57.1 ± 6.6 bA	71.2 ± 7.4 aA
T. harzianum + B. amyloliquefaciens	70.9 ± 2.9 abA	82.5 ± 5.9 aA
**F-value (IxF)**	3.679	
**P-value (IxF)**	0.0284 *	

**Total Mn Accumulation (mg plant^{-1})**		
	**P – fertilization (F)**	
**Inoculation (I)**	100%	60%
Control (no inoculation)	2.03 ± 0.46 bA	1.80 ± 0.32 bA
Trichoderma harzianum	3.85 ± 0.37 aA	3.80 ± 0.21 aA
Bacillus amyloliquefaciens	2.90 ± 0.34 abA	3.15 ± 0.42 abA
T. harzianum + B. amyloliquefaciens	3.55 ± 0.42 aA	4.22 ± 0.30 aA
**F-value (IxF)**	0.633	
**P-value (IxF)**	0.6017 ns	

**Total Cu Accumulation (mg plant^{-1})**		
	**P – fertilization (F)**	
**Inoculation (I)**	100%	60%
Control (no inoculation)	0.57 ± 0.10 bB	1.11 ± 0.03 aA
Trichoderma harzianum	1.33 ± 0.16 aA	1.38 ± 0.17 aA
Bacillus amyloliquefaciens	1.83 ± 0.26 aA	1.53 ± 0.31 aA
T. harzianum + B. amyloliquefaciens	1.61 ± 0.07 aA	1.72 ± 0.13 aA
**F-value (IxF)**	2.078	
**P-value (IxF)**	0.1338 ns	

**Total Zn Accumulation (mg plant^{-1})**		
	**P – fertilization (F)**	
**Inoculation (I)**	100%	60%

Control (no inoculation)	1.56 ± 0.26 bA	1.48 ± 0.26 bA
Trichoderma harzianum	2.46 ± 0.29 aA	2.26 ± 0.09 aA
Bacillus amyloliquefaciens	2.39 ± 0.14 aA	2.11 ± 0.24 abA
T. harzianum + B. amyloliquefaciens	2.46 ± 0.15 aA	2.21 ± 0.13 abA
**F-value (IxF)**	0.083	
**P-value (IxF)**	0.9685^{ns}	

Note: *: $p < 0.05$, **: $p < 0.01$, ns: not significant. Different lowercase letters indicate significant differences among inoculation treatments within the same P rate, and different uppercase letters indicate significant differences among P rates within the same inoculation treatment, according to Tukey's test (0.05 probability).
